# Nutritional Interventions to Improve Clinical Outcomes in Ovarian Cancer: A Systematic Review of Randomized Controlled Trials

**DOI:** 10.3390/nu11061404

**Published:** 2019-06-21

**Authors:** Emanuele Rinninella, Anna Fagotti, Marco Cintoni, Pauline Raoul, Giuseppe Scaletta, Lorena Quagliozzi, Giacinto Abele Donato Miggiano, Giovanni Scambia, Antonio Gasbarrini, Maria Cristina Mele

**Affiliations:** 1 UOC di Nutrizione Clinica, Dipartimento di Scienze Gastroenterologiche, Endocrino-Metaboliche e Nefro-Urologiche, Fondazione Policlinico Universitario A. Gemelli IRCCS, Largo A. Gemelli 8, 00168 Rome, Italy; giacintoabeledonato.miggiano@policlinicogemelli.it (G.A.D.M.); mariacristina.mele@unicatt.it (M.C.M.); 2 Istituto di Patologia Speciale Medica, Università Cattolica del Sacro Cuore, Largo F. Vito 1, 00168 Rome, Italy; pauline.raoul1@gmail.com (P.R.); antonio.gasbarrini@unicatt.it (A.G.); 3 UOC di Ginecologia Oncologica, Dipartimento di Scienze della Salute della Donna e del Bambino e di Sanità Pubblica, Fondazione Policlinico Agostino Gemelli IRCCS, Largo A. Gemelli 8, 00168 Rome, Italy; anna.fagotti@unicatt.it (A.F.); giuscaletta@gmail.com (G.S.); lorenaquagliozzi@gmail.com (L.Q.); giovanni.scambia@policlinicogemelli.it (G.S.); 4 Istituto di Clinica Ostetrica e Ginecologica, Università Cattolica del Sacro Cuore, Largo F. Vito 1, 00168 Rome, Italy; 5 Scuola di Specializzazione in Scienza dell’Alimentazione, Università di Roma Tor Vergata, Via Montpellier 1, 00133 Rome, Italy; marco.cintoni@gmail.com; 6 UOC di Medicina Interna e Gastroenterologia, Dipartimento di Scienze Gastroenterologiche, Endocrino-Metaboliche e Nefro-Urologiche, Fondazione Policlinico Universitario A. Gemelli IRCCS, Largo A. Gemelli 8, 00168 Rome, Italy

**Keywords:** ovarian cancer, diet, nutrition therapy, nutritional support, randomized controlled trials, personalised medicine

## Abstract

Among all gynaecological neoplasms, ovarian cancer has the highest rate of disease-related malnutrition, representing an important risk factor of postoperative mortality and morbidity. Hence, the importance of finding effective nutritional interventions is crucial to improve ovarian cancer patient’s well-being and survival. This systematic review of randomized controlled trials (RCTs) aims at assessing the effects of nutritional interventions on clinical outcomes such as overall survival, progression-free survival, length of hospital stay (LOS), complications following surgery and/or chemotherapy in ovarian cancer patients. Three electronic bibliographic databases (MEDLINE, Web of Science, and Cochrane Central Register of Controlled Trials) were used to conduct a systematic literature search based on fixed inclusion and exclusion criteria, until December 2018. A total of 14 studies were identified. Several early postoperative feeding interventions studies (*n* = 8) were retrieved mainly demonstrating a reduction in LOS and an ameliorated intestinal recovery after surgery. Moreover, innovative nutritional approaches such as chewing gum intervention (*n* = 1), coffee consumption (*n* = 1), ketogenic diet intervention (*n* = 2) or fruit and vegetable juice concentrate supplementation diet (*n* = 1) and short-term fasting (*n* = 1) have been shown as valid and well-tolerated nutritional strategies improving clinical outcomes. However, despite an acceptable number of prospective trials, there is still a lack of homogeneous and robust endpoints. In particular, there is an urgent need of RCTs evaluating overall survival and progression-free survival during ovarian oncology treatments. Further high-quality studies are warranted, especially prospective studies and large RCTs, with more homogeneous types of intervention and clinical outcomes, including a more specific sampling of ovarian cancer women, to identify appropriate and effective nutritional strategies for this cancer, which is at high risk of malnutrition.

## 1. Introduction

Ovarian cancer is one of the most common cancers worldwide with an estimated global incidence in 2018 of 295,414 new cases [1]. Ovarian cancer has the highest mortality rate of all gynaecologic cancers [2] with a poor five year survival rate. Older women are more likely to be initially diagnosed with advanced disease, peaking in the seventh decade of life [3]. Due to the non-specific nature of symptoms (dyspepsia, nausea, lack of appetite, fatigue, abdominal pain), patients may often be found to have advanced disease with a wide intra-abdominal spread of neoplasms at the time of diagnosis. One of the detrimental effects of the involvement of the intra-abdominal gastro-enteric apparatus is a progressive reduction in caloric intake [4], contributing to the impairment of nutritional status and body composition. As a consequence, women with this malignancy tend to have a higher incidence of malnourishment at the time of diagnosis [5]. Since oncology treatments, such as surgery and chemotherapy, produce additional adverse effects such as short bowel, diarrhoea, malabsorption and fatigue [6], malnutrition in these cancer patients has a multifactorial origin and unfortunately is not often recognized [7]. 

Among gynecologic malignancies, the prevalence of malnutrition is higher in ovarian cancer, reaching 70% in some reports [4,8]. Many laboratory and clinical tools have been employed to identify malnutrition in this setting, such as prealbumin or albumin [4,9,10], Nutritional Risk Score (NRS) [8], Subjective Global Assessment (SGA) [11], and bioelectrical impedance analysis (BIA)-derived phase angle [12]. These scores are able to predict longer hospital stays and impaired therapeutic outcomes in malnourished patients [4,9,10,11,12]. 

All these findings underline the problem of malnutrition in ovarian cancer patients and highlight the crucial need to propose adequate nutritional support in order to optimize their wellbeing and maximize their ability to complete cancer treatments and consequently improve survival. 

In recent years, a growing number of randomized clinical trials (RCTs) evaluated the effect of nutritional support on clinical outcomes. This systematic review aims at examining the impact of several types of nutrition interventions on clinical outcomes in ovarian cancer patients.

## 2. Materials and Methods

This systematic review was performed according to the Cochrane Handbook for systematic reviews [13] and followed the Preferred Reporting Items for Systematic Reviews and Meta-Analyses (PRISMA) statement [14].

### 2.1. Eligibility Criteria 

#### 2.1.1. Studies 

Eligible study designs included RCTs including cross-over and parallel designs. 

#### 2.1.2. Participants

Eligible patients must (i) be at least 18 years old with any nutritional status (well-nourished, malnourished, at risk of malnutrition) and (ii) have a histological diagnosis of epithelial ovarian cancer having completed or not primary treatment (surgery, chemotherapy). Due to the limited number of studies carried out on ovarian cancer patients only, we also considered studies on patients with ovarian cancer and other gynecological cancers.

#### 2.1.3. Interventions

Studies with nutritional interventions including nutrition assessment, nutrition counseling, supplementary food or drink, fortified foods, oral nutrition supplements, enteral or parenteral feeds during chemotherapy or during the perioperative period were considered for inclusion in this review. There were no restrictions on frequency, duration, and intensity of interventions. There was also no limitation on intervention settings (e.g., hospital-based, home-based, individual/group counseling). 

#### 2.1.4. Outcomes

The outcomes of interest were overall survival, progression-free survival (PFS), length of hospital stay (LOS), postoperative complications, anthropometry, and quality of life (QoL) measures, following nutritional strategies.

### 2.2. Information Sources and Search Strategy 

#### 2.2.1. Electronic Searches

The search was carried out on 17 December 2018 using three electronic databases MEDLINE (via PubMed), ISI Web of Science and Cochrane Central Register of Controlled Trials. The search strategy was limited to English language articles and there were no restrictions on date of publication. Databases were screened for search terms in titles and abstracts. The search string for each database is described in Table 1.

#### 2.2.2. Hand Searches

Checking reference lists was used to supplement electronic searching. The reference lists of retrieved articles were manually scrutinized to identify potentially relevant studies.

### 2.3. Study Selection

The study selection process was independently carried out by three reviewers (P.R; E.R; M.C). All articles generated from the electronic search were imported into Mendeley^©^ (Elsevier, Amsterdam, The Netherlands), a reference management software, and duplicates were removed. Titles and abstracts of all records were screened for eligibility based on inclusion criteria. All titles assessed as ineligible were excluded. Differences in judgment during the selection process between the three reviewers were settled by discussion and consensus.

### 2.4. Data Extraction 

Information was collected using an Excel^©^ (Microsoft Office, Redmond, WA, USA) spreadsheet specifically developed for this study. Each full-text article was retrieved, and the articles deemed ineligible were excluded and the reasoning reported. Differences in judgment between two reviewers were settled by discussion and consensus.

### 2.5. Risk of Bias and Quality Assessment 

The risk of bias instruments was used for randomized controlled trials and non randomized comparative studies. Based on Cochrane’s guideline, risk of bias was independently assessed by at least two reviewers, then the agreed assessment was further entered into the software Review Manager 5.3.5 (The Nordic Cochrane Centre, Copenhagen, Denmark). 

The articles were assessed as high, low, or unclear risk using recommendations for judging the risk of bias of the “Cochrane Handbook for Systematic Reviews of Interventions”. In total, there are seven domains for quality assessment: (1) Random sequence generation; (2) Allocation concealment; (3) Blinding of participants and personnel; (4) Blinding of outcome assessment; (5) Incomplete outcome data; (6) Selective reporting; (7) Other bias (other source of bias could put the study at a high risk of bias in certain circumstances, e.g., carry-over in cross-over trials, baseline imbalance). Each judgment has three options: low risk, high risk, and unclear risk.

### 2.6. Data Synthesis

Because of the high heterogeneity of the studies’ measures and the types of nutritional intervention, a systematic review was performed. Indeed, the measurement units, nutritional and control interventions of each study were not comparable and consequently, meta-analysis was unfeasible. The main results of the review were displayed on a summary of findings table. For each study, a description of the population, type of intervention, outcomes measures and results were presented. 

## 3. Results

### 3.1. Study Selection

**Figure 1:**
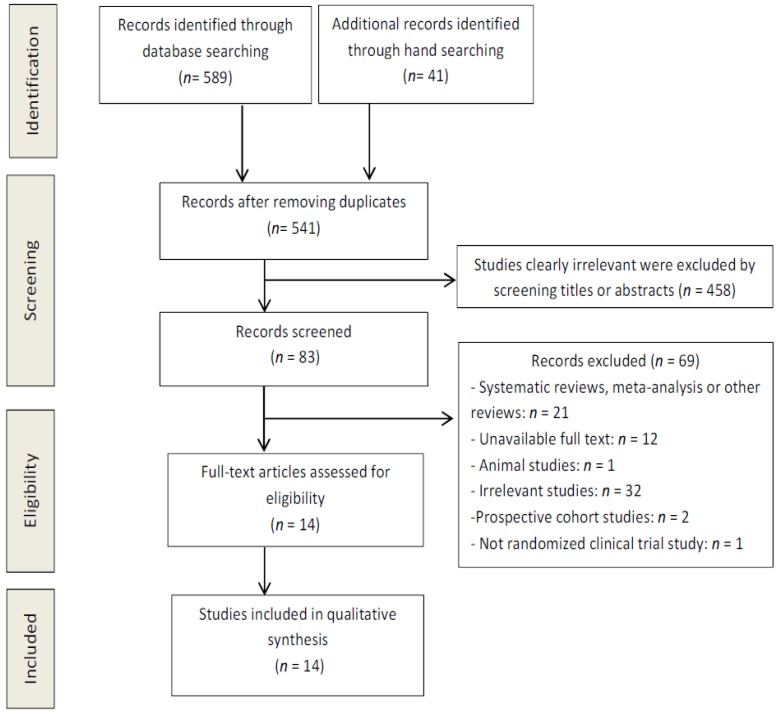
Preferred Reporting Items for Systematic Reviews and Meta-Analyses (PRISMA) 2009 flow diagram.

The flow diagram in Figure 1 displays the results of the literature search and study selection process.

Fourteen studies were identified for inclusion in the systematic review. Following the initial review of titles and abstracts and after hand searching, it was noted that the search strategy had not been sufficiently broad to identify all available studies in this area. Hand searching allowed the identification of fivesignificant studies. 

### 3.2. Study Characteristics

Detailed study characteristics were retrieved in the Table 2.

### 3.3. Study Quality Assessment

**Figure 2:**
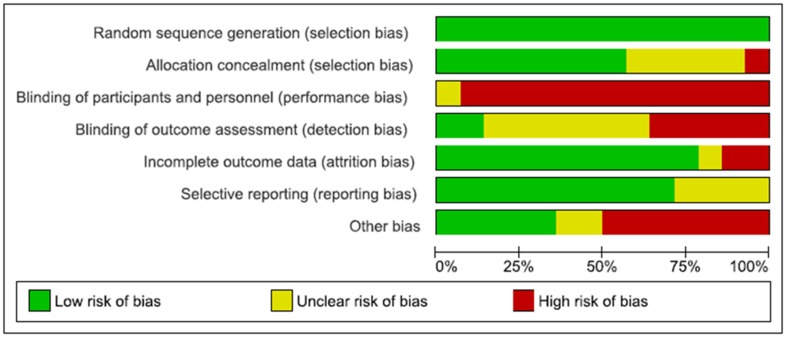
Risk of bias graph: review authors’ judgements about each risk of bias item presented as percentages across all included studies.

The quality of the included studies was assessed in terms of risk of bias. The risk of bias across all included studies is shown in Figure 2.

**Figure 3:**
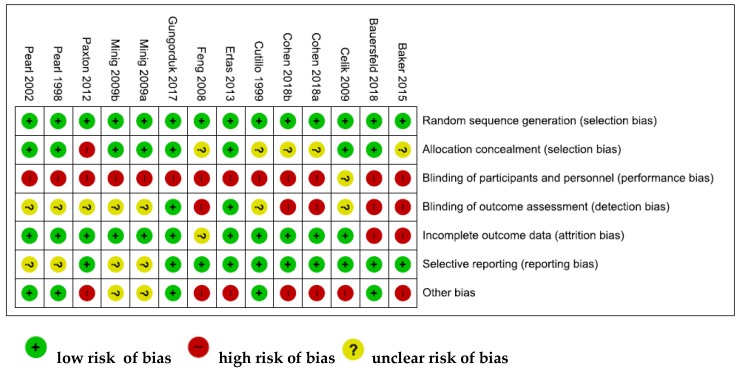
Risk of bias summary: review authors’ judgments about each risk of bias item for each included study.

The low risk of bias (above 50% of the studies) was due to adequate random sequence generation and allocation concealment (selection bias), sufficient outcome data (attrition bias), adequate reporting of results (reporting bias). Regarding other bias, the sample size calculation was not performed in five studies, reducing the validity of their results. Blinding of participants (performance bias) was the highest risk of bias. Indeed, as the nature of the intervention was nutritional, it was not possible to blind participants and study personnel. Although high risk of bias was identified in six out of seven domains across all included studies, the magnitude is relatively small. The risk of bias summary, including an assessment of each risk item in each study, is shown in Figure 3.

None of the studies was assessed as fully low risk of bias. In seven studies, more than half of the domains were assessed to be as low risk of bias [15,16,17,19,22,24,25].

### 3.4. Summary of Findings

#### 3.4.1. Effects of Nutritional Interventions on Overall Survival

Only one RCT evaluated the impact of a post-diagnosis nutritional intervention on overall survival of patients with ovarian cancer. Celik et al. [19] compared immune-enhancing enteral nutrition (IEN) with standard enteral feeding in oncologic gynecologic patients (32% of ovarian cancer patients) after gynecologic oncologic surgery. This study demonstrated no significant differences in terms of mortality rate (*p* > 0.05) between the two groups.

#### 3.4.2. Effects of Nutritional Interventions on LOS

Nine RCTs evaluated the relationship between nutritional interventions and LOS of patients with ovarian cancer.

Non-conventional postoperative feeding interventions were compared with postoperative traditional oral diet in gynecological patients including ovarian cancer after oncologic surgery [15,16,17,19,20,21,23]. In three RCTs [15,20,21], LOS of patients receiving early oral feeding (EOF) was shorter than LOS of patients receiving traditional oral feeding (TOF). Indeed, in the first RCT of Minig et al. [20], LOS was 6.9 ± 2.6 days in the EOF group versus (vs.) 9.1 ± 4.5 days in the TOF group (*p* = 0.022). In the second study of Minig et al. [21], the reduction of LOS was confirmed with 4.7 ± 1.9 days in the EOF group vs 5.8 ± 2.3 days in the TOF group (*p* = 0.006). Finally, Pearl et al. [15] demonstrated that LOS was significantly longer in the TOF group (4.6 ± 2.1 days in the EOF group vs. 5.8 ± 2.7 days in the TOF group; *p* = 0.001). Cutillo et al. [16] developed a RCT comparing, in ovarian (48.3%) and other gynecologic cancer patients, early oral feeding (EOF) with nasogastric decompression followed by feeding at the first passage of flatus, after major oncologic gynecologic surgery. Patients receiving an EOF had a significantly shorter postoperative LOS than patients receiving a nasogastric decompression followed by feeding at the first passage of flatus (median 5 days, range 3–18 vs. median 6 days, range 4–18; *p* < 0.05). However, the study of Baker et al. [23] did not find any significant differences in LOS between ovarian cancer patients receiving postoperative early enteral feeding and patients receiving a standard diet. Pearl et al. [17] showed a comparable LOS between gynecologic cancer patients (including 33% of ovarian cancer patients) who received a regular diet on the first postoperative day compared with patients who received a clear liquid diet as the first postoperative meal. Finally, the use of immune-enhancing enteral nutrition (IEN) was compared with standard enteral feeding in oncologic gynecologic patients (including 32% of ovarian cancer patients) after abdominal surgery [19]. LOS was significantly shorter in IEN patients than standard enteral feeding patients (4.1 ± 1.3 days vs. 7.8 ± 1.2 days; *p* < 0.05) [19].

Ertas et al. [22] evaluated an innovative postoperative gum-chewing intervention (30 min of chewed gum, three times/day, from the first operative morning until the first passage of flatus) on LOS of patients undergoing abdominal complete surgical staging for various gynecological cancers including ovarian cancer. This study demonstrated that LOS was significantly reduced in patients that chewed gum compared with controls (5.9 ± 1 vs. 7.0 ± 1.4 days; *p* < 0.001) [22]. Moreover, the impact of coffee consumption was evaluated by Güngördük et al. [24]. This RCT showed that coffee consumption during the early postoperative period after abdominal surgery reduces LOS (7.4 ± 2.9 days in the control group vs. 6.1 ± 1.1 in the coffee group; *p* = 0.003).

#### 3.4.3. Effects of Nutritional Interventions on Postoperative Clinical Outcomes

Nine studies evaluated the effect of nutritional interventions on postoperative functional activity recovery of gynecologic cancer patients (including ovarian cancer) undergoing abdominal surgery such as time to bowel motility (bowel sounds, flatus elimination, passage of stools), analgesic and antiemetic requirements, time before solid diet tolerated, postoperative nausea and vomiting.

Patients who received the early clear liquid diet showed faster recovery of postoperative intestinal function in terms of time to bowel sounds and time to flatus [15,20,21] compared to control group (clear liquid diet on the first postoperative day or nothing by mouth until the resumption of normal bowel function). The time to development of bowel sounds (1.2 ± 0.5 days) and passage of flatus (2.8 ± 1.4 days) were comparable between patients receiving a regular diet on the first postoperative day and patients receiving a clear liquid diet as the first postoperative meal [17]. The incidence of postoperative nausea in the early clear liquid diet feeding was nearly twice that of the traditional feeding group (43.5% in the EOF group vs. 24.3% in the TOF group; *p* = 0.006) [15], whereas the frequency of vomiting were comparable in both groups [15,21]. The incidence of nausea/ vomiting was comparable between ovarian cancer patients who received a regular diet on the first postoperative day and patients who received a clear liquid diet as the first postoperative meal [17]. In two RCTs [15,21], the time to tolerance of solid diets was shorter in the early clear liquid diet group compared to the traditional group. Additional requirements of analgesic and antiemetic drugs were similar between the early clear liquid diet group and the traditional group [20,21]. The parallel design RCT of Cutillo et al. [16] demonstrated that EOF reduced postoperative discomfort and allowed a more rapid recovery in gynecologic patients (48.3% of ovarian cancer) [16]. Indeed, patients receiving an EOF were associated with a significantly faster resolution of postoperative ileus (*p* < 0.01), a more rapid return to a regular diet (3 days, range 2–14 vs. 5 days, range 2–8; *p* < 0.01) and an earlier first passage of stool (3 days, range 1–10 vs. 4 days, range 1–8; *p* < 0.01) and flatus (2 days, range 1–4 vs. 3 days, range 1–6) than patients receiving a nasogastric decompression. Nevertheless, rates of nausea and vomiting were similar in both conditions. Moreover, Feng et al. [18] conducted a RCT comparing a semiliquid diet with clear feeds, both started at 6 h in oncologic gynecologic patients (18.3% of ovarian cancer patients) after major abdominal gynecological oncology surgery. There were significantly higher incidences of nausea (17 patients vs. 7 patients; likelihood ratio *χ*^2^ = 6.944; *p* = 0.008) and a shorter time for regular diet resumption in patients with semiliquid diet than in those with clear feeds (*t* = 4.112; *p* = 0.000) [18]. No significant differences were found in vomiting, in time to development of bowel sound and in passage of flatus [18].

Other interventions such as gum-chewing [22] and coffee consumption [24] after surgery were explored as adjuvant tools to improve bowel motility (first bowel movement, flatus, defecation time), and the ability to tolerate food. Moreover, gum-chewing intervention significantly reduced analgesic (relative risk RR = 7.8; 95% confidence interval CI: 1.0–61.5; *p* = 0.03) and antiemetic requirements (RR = 4.8; 95% CI: 1.0–21.1; *p* = 0.03) compared to the control group. 

#### 3.4.4. Effects of Nutritional Interventions on Postoperative Complications

Regarding early postoperative feeding interventions, four RCTs assessed the impact of early postoperative clear liquid diet feeding on the first postoperative day, compared to traditional oral feeding. Two RCTs [15,20] observed a nonsignificant difference in the incidence of overall, infective or intestinal complications between early clear diet group and traditional group. On the other hand, Minig et al. performed another RCT [21] demonstrating significantly higher overall postoperative complications in patients who received TOF than in patients receiving EOF (39% vs. 17% in the EOF group; *p* = 0.003). Indeed, in this RCT, infective postoperative complications were significantly higher in the TOF group (14% vs. 3%; *p* = 0.017) and the occurrence of postoperative ileus was higher in the TOF than in the EOF branch, although this was not statistically significant (6% vs. 1%; *p* = 0.367). Additionally, two patients in the TOF group required readmission (for fever syndrome and postoperative ileus) vs. none in the EOF group [21]. Celik et al. [19] demonstrated that perioperative immunonutrition decreased postoperative complications in patients undergoing surgery for gynecological malignancy (including 32% of ovarian cancer patients) by increasing the immunologic response. Regarding the influence of gum-chewing, the occurrence of mild ileus symptoms was higher in patients in the control group compared to patients in the gum-chewing group (RR = 2.4; 95% CI: 1.2–4.5; *p* = 0.004) [22]. 

#### 3.4.5. Effects of Nutritional Interventions on Dietary Intake and Anthropometric Measures

Few studies evaluated the effect of different dietary prescriptions on patients’ dietary intake and body weight or composition. Baker et al. reported no significant differences in protein and energy intake and in weight increase between early postoperative enteral feeding group and traditional diet group [23]. The parallel-arm RCT of Cohen et al. [26] compared the body composition of gynecologic cancer patients (62.2% of ovarian cancer patients) assigned to the diet recommended by the American Cancer Society (ACS) with the body composition of gynecologic cancer patients assigned to a ketogenic diet (KD). This study demonstrated that the KD, compared with ACS diet, produced significantly lower levels of adjusted total (35.3 kg compared with 38.0 kg; *p* < 0.05) and android (3.0 kg compared with 3.3 kg; *p* < 0.05) fat mass. Paxton et al. found a significantly higher fiber intake (+5.2 g/day), daily servings of juice (+0.9 servings/day) and vegetables (+1.3 servings/day) (all *p* < 0.05) in ovarian cancer survivors consuming a low fat/high fiber (LFHF) diet compared with the arm consuming fruit and vegetable juice concentrates (FVJC) diet [28]. However, no significant weight changes were appreciated between the two groups [28].

#### 3.4.6. Effects of Nutritional Interventions on QoL

The use of both an early postoperative enteral feeding [23], and an early postoperative resumption of oral intake with a clear liquid diet [20,21] did not significantly improve patients’ well-being compared to standard of care. Pain and QoL scores did not differ significantly between the groups [21]. The impact of a 6 month dietary intervention (LFHF diet or FVJC diet) in ovarian cancer survivors was studied by Paxton et al. [28]: in their study, no significant differences in Health-related QOL were observed between LFHF group and FVJC group [28]. Cohen et al. [27] compared the impact of the diet recommended by the ACS diet with a KD in patients with ovarian or endometrial cancer. Compared to the ACS, the KD group improved perceived physical functional status as well as reduced cravings for starchy food and fast food fats (*p* < 0.05 for both). These findings suggest that a KD is feasible for ovarian cancer patients and may provide several benefits that improve QoL [27]. A recent cross over pilot RCT [25] studied the effects of short-term fasting (STF)—36 h before and 24 h after chemotherapy (60 h-fasting period) including a maximum total daily energy intake of 350 kcal on QoL and tolerance to chemotherapy in patients with breast and ovarian cancer (11.7%). This study reported beneficial effect of STF on QoL and fatigue during chemotherapy, in particular when STF was proposed in the first half of chemotherapies cycles. Indeed, the chemotherapy-induced reduction of QoL was less than the minimally important difference (MID; Functional Assessment of Cancer Therapy—General (FACT-G) = 5) for STF, but greater than the MID for non-fasted periods.

## 4. Discussion 

Disease-related malnutrition is well known to be an important risk factor of postoperative mortality and morbidity in ovarian cancer patients [11,12]. As weight loss reduces the ability of patients to be effectively treated with chemotherapy [29], the more oncologic patients are well-nourished by receiving adequate nutritional support by clinicians, the better they are able to optimally withstand cancer treatments.

This systematic review aimed at assessing whether nutritional interventions could improve clinical outcomes, such as overall survival, PFS, LOS, clinical conditions and complications following surgery, anthropometry and QoL measures in ovarian cancer patients during chemotherapy, or during the perioperative period. Only RCTs were considered in order to conduct a meta-analysis and thus obtain clear and valid conclusions, but all the reviewed RCTs were based on heterogeneous clinical outcomes, used different nutritional interventions and control groups, thus resulting not suitable for a meta-analysis.

The majority of RCTs reported improved clinical outcomes after nutritional interventions [15,16,18,19,20,21,22,24,26,27,28]. Most of them found a reduction in LOS and ameliorated intestinal recovery after surgery [15,16,19,21,22,24]. In particular, patients receiving postoperative early enteral feeding had significantly accelerated intestinal recovery following surgery [15,16,21]. Moreover, inexpensive nutritional strategies such as chewing gum use [22] and coffee consumption [24] in the postoperative period have been shown as valid and well-tolerated tools to accelerate intestinal recovery in gynecologic oncology patients, including ovarian cancer patients. Additionally, a parallel trial [28] comparing a low-fat high-fiber diet (LFHF) to a fruit and vegetable juice concentrate (FVJC) reported an improved energy intake in the LFHF group. Finally, up to now, only one trial [27] demonstrated that KD did not negatively affect QoL (physical function, perceived energy), and that it may decrease food cravings and induce selective fat mass loss without lean mass reduction in ovarian or endometrial cancer patients. 

The major strength of this review lies on the gathering of the latest researches on all the nutritional strategies, including innovative nutritional interventions, in order to improve clinical outcomes in ovarian cancer patients. Since the correlation between malnutrition, disease complications and morbidity in oncology patients was demonstrated in different clinical settings [30,31,32,33], nutritional and dietary management progressively have been under the spotlight in the field of gynecologic malignancies in the last decade [8,11,34,35]. However, until now, the number of reviews on this topic specifically addressing ovarian cancer remains very limited. Indeed, in recent years, only one review [36] evaluated the impact of perioperative traditional early vs delayed oral fluids and food, reporting a reduction of complications and LOS in ovarian cancer patients. The authors were unable to identify any RCTs evaluating the use of nutrition supplementation or nutritional counselling in women with ovarian cancer [36] and our review agrees with their conclusions. Indeed, due to several limitations, we also cannot reach clear conclusions despite the rigorous methodology adopted. Nevertheless, our review highlighted a few innovative nutritional approaches, such as high fibre diet, fruit and vegetable juice concentrate supplementation [28], ketogenic diet intervention [27,28], coffee [24] and chewing-gum [22] consumptions.

Furthermore, this review highlighted an intriguing crossover pilot study [25] which measured the effect of STF during chemotherapy and reported improved tolerance to the treatment and QoL among gynecologic patients (including 11.7% ovarian cancer patients). The scientific rationale of this study relies on the results of in vitro and animal studies showing cancer cells sensitization to chemotherapy after fasting for 48 h [37,38]. There is a growing interest on the effect of STF or fast-mimicking diets (FMDs) during chemotherapy [39,40] and many clinical trials on this topic are ongoing [41,42,43,44,45,46,47]. However, these results should be taken with extreme caution [48]. Indeed, considering the lack of strong clinical data (RCTs or prospective cohort studies with solid endpoints) on FMDs or STF, a position paper from the Italian Society of Medical Oncology (AIOM) and the Italian Society of Artificial Nutrition and Metabolism (SINPE) raised concerns particularly for malnourished patients [49]. Further evidences are required to confirm the effects of STF and identify the right set of patients whom this approach could be applied to and could really benefit from it [50].

Several limitations of the evidence shown in this systematic review require acknowledgment. Firstly, only 2 out of 14 [23,28] included exclusively ovarian cancer patients. For this reason, RCTs evaluating all gynecological cancer patients—including ovarian cancer—were also considered during the data selection process. The majority of the reviewed RCTs used a mixed sample of gynecological cancer patients and, in 8 out of 12 studies, less than 50% of the patients’ samples were ovarian cancer [15,16,17,18,19,22,24,25]. Moreover, all these studies—in which ovarian cancer patients were categorised as gynaecological cancer patients—did not report separate outcomes according to cancer type, making it difficult to assess whether the results were consistent with ovarian cancer patients. Secondly, the small sample sizes and the variations in cancer stage, treatment history, and concurrent chemotherapy status of participants may also influence the results and represent a potential risk of bias. Thirdly, only 7 out of 14 RCTs performed a sample size calculation [16,20,21,22,24,25,26], making the validity of their findings statistically reliable. Another limitation is the high risk of performance bias of all included studies. Indeed, it was not feasible to blind the participants due to the nutritional intervention received and it may influence some nutritional intervention effect estimates. 

## 5. Conclusions

Our systematic review highlighted the absence of data regarding the effect of nutritional interventions on PFS during ovarian oncology treatments. Only one study [19] evaluated the effect of immunonutrition on mortality rate. Regarding ovarian cancer, the importance to find nutritional interventions in order to improve patient’s survival is even more crucial since the ovarian mortality rate is one of the highest among malignancies [4,8]. 

Further high-quality studies, especially prospective studies and large RCTs, with more homogeneity among types of intervention and clinical outcomes, including a large number of ovarian cancer women, are required to propose new nutritional strategies and to investigate the effect of such strategies as possible modifiers of relevant outcomes such as ovarian cancer survival and PFS.

## Figures and Tables

**Table 1:** Full search strategies for three databases.

**MEDLINE**	
**Set**	**Search Terms**
1	diet*[TextWord] OR diet therapy[MeSHTerms] OR therapy nutrition[MeSHTerms] OR eat*[TextWord] OR food*[TextWord] OR feed*[TextWord] OR meal*[TextWord] OR nutriment*[TextWord] or nutritional advice*[TextWord] OR nutritional counseling*[TextWord] OR nutritional support*[TextWord] OR nutritional intervention*[TextWord]
2	ovar* [TextWord] OR ovary [MeSHTerms]
3	cancer*[TextWord] OR oncology[TextWord] OR tumour*[TextWord] OR tumor*[TextWord] OR malignan*[TextWord] OR carcinoma[TextWord] OR neoplasm*[TextWord]
4	2 AND 3
5	1 AND 4
6	5 and Refined by: Publication Type: “Randomized Controlled Trial”
7	6 and Refined by: Subjects: “Humans”
**WEB OF SCIENCE**	
**Set**	**Search Terms**
1	Topic: (diet* OR eat* OR food* OR feed* OR meal* OR nutriment* or nutritional advice* OR nutritional therapy* OR nutritional support* OR nutritional intervention*)
2	Topic: (Ovarian OR Ovary)
3	Topic: (cancer* OR oncology OR tumour* OR tumor* OR malignan* OR carcinoma OR neoplasm*)
4	1 AND 2 AND 3
5	#4 AND Refined by: Topic: (randomised controlled trial* OR randomised controlled clinical trial* OR randomised controlled study OR randomised controlled clinical study OR randomized controlled trial* OR randomized controlled clinical trial* OR randomized controlled stud* OR randomized controlled clinical stud* OR randomised-controlled trial* OR randomised-controlled clinical trial* OR randomised-controlled study OR randomised-controlled clinical study OR randomized-controlled trial* OR randomized-controlled clinical trial* OR randomized-controlled stud* OR randomized-controlled clinical stud*)
**COCHRANE**	
**Set**	**Search Terms**
1	Title Abstract Keyword: diet* OR eat* OR food* OR feed* OR meal* OR nutriment* or nutritional advice* OR nutritional therapy* OR nutritional support* OR nutritional intervention*
2	Title Abstract Keyword: ovarian OR ovary
3	Title Abstract Keyword: cancer* OR oncology OR tumour* OR tumor* OR malignan* OR carcinoma OR neoplasm*
4	1 AND 2 AND 3

Abbreviation: * star search algorithm.

**Table 2:** Characteristics of included studies.

Study ID	Study Design	Cancer Types with % Ovarian Cancer	Sample Size	Time of Intervention	Type of Nutritional Intervention	Comparison	Outcomes Measures	Results
Pearl et al. 1998 [15]	RCT	Ovarian (32.3%), cervical, uterine and benign cancers	*n* = 195	Post-operation on the first postoperative day	EOF: Clear liquid diet	TOF: Nothing by mouth until bowel sounds, the passage of stool or flatus	Incidence of vomiting and nausea Intestinal function recovery LOS Postoperative complications	-No significant differences between the two groups in: incidence of vomiting postoperative complications -Significantly more patients in the EOF group developed nausea (*p* = 0.006) -Significantly reduction in the EOF group of: time of development of bowel sounds (*p* = 0.007) time to initiation of clear liquid and regular diets (*p* < 0.001) LOS (*p* = 0.001)
Cutillo et al. 1999 [16]	RCT with parallel arm design	Ovarian (48.3%) and other gynecologic cancers	*n* = 122	Post-operation on the first postoperative day	EOF: Clear-fluid diet, passing to a semiliquid fiberless diet within the next 24 h	Nasogastric decompression followed by feeding at the first passage of flatus	Incidence of nausea and vomiting Intestinal function recovery: time to first passage of flatus and stool, time elapsed before adequate tolerance of a regular diet LOS Postoperative complications	-No significant differences between the two groups in incidence of nausea and vomiting. -Significant reduction in the EOF group of: time of resolution of postoperative ileus(*p* < 0.01) time elapsed to a regular diet (*p* < 0.01) time of first passage of stool (*p* < 0.01) LOS (*p* < 0.05)
Pearl et al. 2002 [17]	RCT	Ovarian (33%), cervical, uterine and benign cancers	*n* = 245	Post-operation on the first postoperative day	EOF: Regular diet	EOF: Clear liquid diet	Incidence of nausea and vomiting Abdominal distention, passage of flatus before discharge LOS Postoperative complications	-No significant differences between the two groups in: incidence of nausea and vomiting abdominal distention time of first passage of flatus before discharge LOS postoperative complications
Feng et al. 2008 [18]	RCT	Ovarian (18.3%) and other gynecologic cancers	*n* = 60	Post-operation on the first 6 postoperative hours	Semiliquid diet followed by regular diet	Clear-liquid diet tosemiliquid diet toregular diet	Incidence of nausea and vomiting Time to development of bowel sound and passage of flatus Pre and post operative weight Urine acetone Fasting blood sugar	-Significant reduction (*p* < 0.05) in clear feeds group of: incidence of nausea time of regular diet resumption -No significant differences between the two groups in: incidence of vomiting time to development of bowel sound time of first passage of flatus pre and post operative weight urine acetone and fasting blood sugar
Celik et al. 2009 [19]	RCT with parallel arm design	Ovarian (32%) and other gynecologic cancers	*n* = 50	Pre-operation on the last 2 preoperative days post-operation on the first 7 postoperative days	IEN	Standardenteral nutrition	Nutritional measures (albumin, prealbumin) LOS Postoperative complications Mortality rate.	-No significant differences between the two groups in: nutritional measures mortality rate (*p* > 0.05) -Significant reduction in patients receiving IEN vs patients receiving standard enteral nutrition in: LOS (*p* < 0.05) postoperative complications (*p* < 0.05) for wound infections and dehiscence
Minig et al. 2009a [20]	RCT	Ovarian cancer (87.5%)	*n* = 40	Post-operation during the first 24 postoperative hours	EOF: Clear liquid diet	TOF: Nothing by mouth until the resumption of normal bowel function	LOS VAS score (abdominal pain) QoL (EORTC OV-28 and EORTC C-30) EBL Incidence of nausea and vomiting Postoperative complications Intestinal function recovery Analgesic and antiemetic drugs requirements	-Significant reduction of LOS (*p* = 0.022) in EOF group vs TOF group. -No significant differences between both groups in: VAS score QoL EBL incidence of nausea and vomiting postoperative complications intestinal activity recovery analgesic and antiemetic drugs needs
Minig et al. 2009b [21]	RCT	Ovarian (58%), endometrial, cervix and other cancers	*n* = 143	Post-operation during the first 24 postoperative hours	EOF: Clear liquid diet	TOF: Nothing by mouth until the resumption of normal bowel function	LOS VAS score (Abdominal pain) QoL Incidence of nausea and vomiting Postoperative complications Intestinal function recovery Analgesic and antiemetic drugs requirements	-Significant reduction of LOS in the EOF group (*p* = 0.006). -Significant higher overall postoperative (*p* = 0.003) and infective complications (*p* = 0.017) in the TOF group compared to the EOF group. -Significant higher mean level of postoperative satisfaction (*p* < 0.001) in the EOF group. -No differences between both groups in: QoL incidence of nausea and vomiting abdominal pain analgesic and antiemetic drugs requirements
Ertas et al. 2013 [22]	RCT	Ovarian (36.9%), endometrial and cervix cancers	*n* = 149	Post-operation on the first postoperative morning until the first passage of flatus	Chewing-gum 3 times/day	Control	Postoperative intestinal function recovery LOS	-Significant reduction (*p* < 0.001) in patients who chewed gum compared to controls of: time to flatus and defecation time to bowel movement time to tolerate diet LOS
Baker et al. 2015 [23]	RCT	Ovarian cancer (100%)	*n* = 109	Post-operationon the first postoperative day	Early enteral feeding: standard fiber (20P:30F:50C) 125 kJ/kg body weight.Until adequate oral intake could be maintained: 65–75% of the daily nutritional requirements.	Standard oral diet	QoL: FACT-G, FACT-O, EQ5D index, Euroqol-VAS, ICU or HDU admission %Nausea/vomiting Blood transfusion % Nutritional status (PG-SGA score) Pain score Weight Protein and energy intake LOS	-No significant differences between both groups in: QoL LOS pain score Euroqol-VAS ICU or HDU admission % Nausea/vomiting blood transfusion % protein and energy intake weight -Improvements of nutritional status (PG-SGA score) in the early enteral feeding patients vs. controls: significant different (*p* < 0.05) at 7 days postoperatively only (intention-to-treat analysis).
Güngördüket al. 2017 [24]	RCT	Ovarian (39.5%), endometrial, cervical and fallopian cancers	*n* = 114	Post-operationon the first morning after surgery	3 cups of caffeinated coffee daily (100 mL at 10:00 AM, 3:00 PM and /:00 PM)	Routine care without coffee consumption	Intestinal activity recovery (time to the first passage of flatus after surgery, time to first defecation, time to first bowel movement, time to toleration of a solid diet) LOS	-Significant reduction (*p* < 0.001) in patients who consumed coffee compared with controls in: time to flatus and defecation time to bowel movement time to tolerate diet. -Reduction of LOS in patients who consumed coffee compared to controls.
Bauersfeld et al. 2018 [25]	RCT with cross-over design	Ovarian (11.7%) and breast cancers	*n* = 34	During CT	Group ASTF* of 60 h (36 h before to 24 h after CT) during the first three of scheduled 6 CTsthenstandard Mediterranean diet during the last three of scheduled 6 CTs.	Group BStandard Mediterranean diet during the first three of scheduled 6 CTsthenSTF* of 60 h (36 h before to 24 h after CT) during the first three of scheduled 6 CTs	QoL: FACT-G Fatigue: FACIT-F	-In the group A, significant improvements during fasted periods compared to standard diet in: QoL fatigue -In the group B, no significant reduction during fasted period compared to standard diet in QoL Fatigue
Cohen et al. 2018 [26]	RCT	Ovarian (62.2%) and endometrial cancers	*n* = 45	During (*n* = 11) or post-CT	KD diet (70:25:5 energy from fat, protein, and carbohydrate)	ACS diet: high fiber, lower fat	Body composition: android fat mass, visceral fat, lean mass Fasting serum insulin	-Significant reduction in the KD group compared to the ACS group of: total and android fat mass (*p* < 0.05) percentage of change in visceral fat (*p* < 0.05) fasting serum insulin (*p* < 0.01) -No significant differences between both groups in adjusted total lean mass.
Cohen et al. 2018 [27]	RCT with parallel arm design	Ovarian (62.2%) and endometrial cancers	*n* = 45	During CT (*n* = 11) or post-CT	KD diet (70:25:5 energy from fat, protein, and carbohydrate)	ACS diet: moderate- to high-carbohydrate, high fiber, low fat	Mental function by Medical Outcomes Study Short Form-12 Health Survey (SF-12 Appetite (VAS) Food cravings by FCI	-No significant differences between both groups in mental function hunger and appetite -Less frequent food cravings in KD group than ACS group at 12 weeks (*p* < 0.05).
Paxton et al. 2012 [28]	RCT with parallel arm design	Ovarian cancer (100%)	*n* = 52	Post-CT≥6 months	LFHF group	FVJC group	Serum carotenoid and tocopherol levels Dietary intake Weight QoL	-Significant improvements in both groups (*p* < 0.01) in: serum carotenoid and alpha-tocopherol levels -Significant improvement in the LFHF group (*p* < 0.05) in: dietary intake (fiber intake, daily servings of juice fruits and vegetables) -No difference in both groups in: QoL Weight

Abbreviations: ACS, american cancer society; BMI, body mass index; C, carbohydrate; EBL, estimated blood loss; ECOG, eastern cooperative oncology group; EOF, early oral feeding; EORTC, European Organization for the Research and Treatment of Cancer; F, fat; FACIT-F, Functional Assessment of Chronic Illness Therapy-Fatigue; FACT-G, Functional Assessment of Cancer Therapy—General; FACT-O, Functional Assessment of Cancer Therapy—Ovarian; FCI, food craving inventory; FVJC, fruit and vegetable juice concentrates; HDU, high dependency unit; ICU, intensive care unit; IEN, immunenhancing enteral nutrition; KD, ketogenic diet; LFHF, low fat high fibre; LOS, length of hospital stay; n, number; P, protein; PGA-SGA score, Patient-Generated Subjective Global Assessment; Post-CT, post-chemotherapy treatment; QoL, quality of life; RCT, randomized controlled trial; STF, short-term fasting; TOF, traditional oral feeding; CT, chemotherapy treatment; VAS, visual analogue scale. * Fasting period: unrestricted amounts of water, herbal tea, 2x100cl vegetable juice and small standardized quantities of light vegetable broth with a maximum total daily energy intake of 350 kcal.
